# Hypomagnesemia Is Associated with Increased Mortality among Peritoneal Dialysis Patients

**DOI:** 10.1371/journal.pone.0152488

**Published:** 2016-03-29

**Authors:** Kedan Cai, Qun Luo, Zhiwei Dai, Beixia Zhu, Jinping Fei, Congping Xue, Dan Wu

**Affiliations:** Department of Nephrology, Ningbo No. 2 Hospital, Ningbo University School of Medicine, Zhejiang, China; Hospital Universitario de La Princesa, SPAIN

## Abstract

**Objective:**

Hypomagnesemia has been associated with an increase in mortality among the general population as well as patients with chronic kidney disease or those on hemodialysis. However, this association has not been thoroughly studied in patients undergoing peritoneal dialysis. The aim of this study was to evaluate the association between serum magnesium concentrations and all-cause and cardiovascular mortalities in peritoneal dialysis patients.

**Methods:**

This single-center retrospective study included 253 incident peritoneal dialysis patients enrolled between July 1, 2005 and December 31, 2014 and followed to June 30, 2015. Patient’s demographic characteristics as well as clinical and laboratory measurements were collected.

**Results:**

Of 253 patients evaluated, 36 patients (14.2%) suffered from hypomagnesemia. During a median follow-up of 29 months (range: 4–120 months), 60 patients (23.7%) died, and 35 (58.3%) of these deaths were attributed to cardiovascular causes. Low serum magnesium was positively associated with peritoneal dialysis duration (r = 0.303, p < 0.001) as well as serum concentrations of albumin (r = 0.220, p < 0.001), triglycerides (r = 0.160, p = 0.011), potassium (r = 0.156, p = 0.013), calcium(r = 0.299, p < 0.001)and phosphate (r = 0.191, p = 0.002). Patients in the hypomagnesemia group had a lower survival rate than those in the normal magnesium groups (p < 0.001). In a multivariate Cox proportional hazards regression analysis, serum magnesium was an independent negative predictor of all-cause mortality (hazard ratio [HR] = 0.075, p = 0.011) and cardiovascular mortality (HR = 0.003, p < 0.001), especially in female patients. However, in univariate and multivariate Cox analysis, △Mg(difference between 1-year magnesium and baseline magnesium) was not an independent predictor of all-cause mortality and cardiovascular mortality.

**Conclusion:**

Hypomagnesemia was common among peritoneal dialysis patients and was independently associated with all-cause mortality and cardiovascular mortality.

## Introduction

Magnesium (Mg), the fourth most abundant cation in the body and the second most abundant cation in the intracellular space, plays an essential role in numerous biological processes, including cardiovascular function. Although hypomagnesemia is known to play a role in the pathogenesis of arterial hypertension, endothelial dysfunction, dyslipidemia, and inflammation [1], little attention has been given to this condition, and magnesium is referred to as the neglected cation. Recently, there has been increased interest in this area, especially regarding the possible relationship between hypomagnesemia and cardiovascular disease (CVD). Hypomagnesemia is significantly associated with an increased risk of mortality in hemodialysis (HD) patients as well as in the general population and patients with predialysis chronic kidney disease (CKD) [2–4].

In 2014, 55,373 patients received peritoneal dialysis (PD) in China. The 5-year survival rates for patients undergoing PD are 67.5% in Japan [5], 69.8% in Korea [6], and 74.4% in China [7]. These rates are far below the mortality rate for the general population, and CVD is the primary cause of mortality among PD patients, accounting for nearly 40% of all deaths in PD patients [7]. Traditional CVD risk factors do not fully explain the increased mortality observed in PD patients. Patients undergoing PD with peritoneal dialysate containing 0.25 mmol/L magnesium were reported to exhibit a considerable decline in serum magnesium levels [8]. However, few studies have examined the relationship between serum magnesium level and the risk of death during PD. The aim of this study was to investigate whether low serum magnesium levels can predict mortality in incident PD patients.

## Methods

### Patients

We studied all patients who used PD as the first renal replacement in our PD center from July 1, 2005 until December 31, 2014 and were followed to June 30, 2015. Patients were excluded for the following reasons: survival less than 3 months following the initiation of PD, recovered renal function, insufficient data, a history of HD before the start of PD, or lack of follow-up. After application of these exclusion criteria, this retrospective observational study included a total of 253 incident PD patients. Patients were dialyzed with a low-magnesium dextrose peritoneal dialysate (containing 0.25 mmol/L Mg^2+^, 1.25 mmol/L Ca^2+^, 132 mmol/L Na^+^, 95 mmol/L Cl^–^) produced by Baxter Healthcare (Guangzhou, China).The study was approved by the Ethics Committee of Ningbo No. 2 Hospital. The patients’ privacy was protected.

### Data collection

Demographic data were collected at the initiation of PD and included age, gender, and body mass index(BMI), etiology of end-stage renal disease, and prevalence of diabetes. Clinical data and biochemical data were obtained in the first 1–3 months of PD. Clinical data included blood pressure, medications, ultrafiltration volume, and urine volume. Laboratory data included serum levels of magnesium, potassium, sodium, hemoglobin, albumin, total cholesterol, urea nitrogen, and creatinine, intact parathyroid hormone(iPTH). Serum magnesium was also collected in the first follow-up year. Change of serum magnesium was determined by △Mg, which was the difference between 1-year magnesium and baseline magnesium. Body mass index was calculated as weight (kg) divided by the square of height (m^2^). Baseline residual renal function(RRF) was assessed by calculating the glomerular filtration rate (GFR) using the Chronic Kidney Disease Residual GFR Epidemiology Collaboration creatinine equation. Adequacy of dialysis (total weekly creatinine clearance) was measured using PD Adequest software. All biochemical tests were conducted in the laboratory of Ningbo No.2 Hospital. Serum magnesium was determined by enzymic method in Siemens ADVIA 2400 automatic biochemical analyzer. The intra-group and inter-group variability are less than 5% and 10% respectively. Authors had access to identify information during or after data collection. All the patient records were anonymized and de-identified before analysis. Hypomagnesemia was defined as a serum magnesium level less than 0.70 mmol/L [9].

### Outcomes

All patients received follow-up until cessation of PD, death, or June 30, 2015. The primary outcome variable was all-cause or cardiovascular mortality. Cardiovascular mortality was defined as death from acute heart failure, myocardial infarction, fatal arrhythmia, stroke, peripheral artery disease, or sudden death [10–11].

### Statistical analysis

All statistical analyses were performed with SPSS software, version 19.0 (SPSS Inc., Chicago, IL, USA). A p value < 0.05 was considered statistically significant. The results were expressed as frequencies and percentages for categorical variables, means with standard deviation (SD) for normally distributed continuous variables, and median values (interquartile ranges) for non-normally distributed continuous variables. Student’s t-test for independent samples was used for normally distributed continuous variables. Comparisons of non-normally distributed continuous variables were performed using the Mann–Whitney U-test. For categorical variables, the chi-square test was used. The bivariate correlation analysis was tested to assess the association between baseline levels of serum magnesium and the demographic and clinical data. The correlations between magnesium levels and other variables were assessed by Pearson’s or Spearman’s correlation analysis.

Survival analysis was performed to assess associations of serum magnesium levels with all-cause and cardiovascular mortalities. Survival times were established from Kaplan–Meier curves, and differences in survival probabilities between groups were assessed using the log-rank test. The associations between serum magnesium levels and all-cause mortality and cardiovascular mortality were examined via Cox proportional hazards models. Covariates with p < 0.05 in the univariate Cox analysis or thought to be related to the magnesium level were chosen for multivariate Cox proportional hazard analysis. The results were expressed as hazard ratios (HRs) and 95% confidence intervals (95% CIs).

## Results

### Baseline patient characteristics

**Fig 1:**
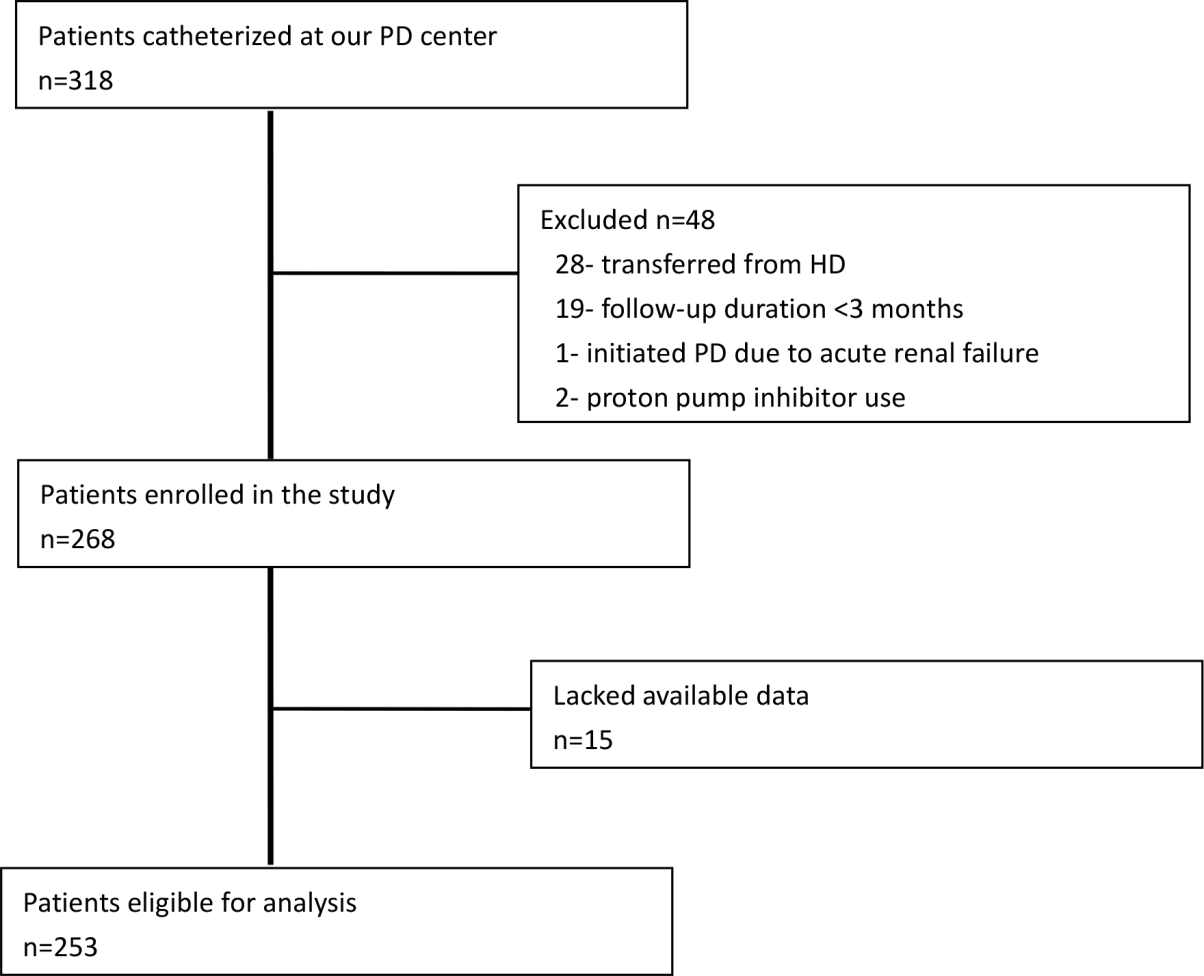
Patient selection scheme.

In total, 318 incident PD patients were catheterized at our PD center during the recruitment period. From these patients, 50 were eliminated because one was undergoing PD due to acute renal failure, 28 were transferred from temporary HD, 19 were on PD less than 3 months, and 2 patients were taking proton pump inhibitors. The remaining 268 patients were enrolled in this study. Of these 268 patients, 253 patients had sufficient available baseline data and were eligible for inclusion in the present analysis (Fig 1). The mean age of these patients was 58±16 years. Also, 55.3% of patients were men, and 22.9% were diagnosed with diabetes mellitus. The leading cause of end-stage renal disease was glomerulonephritis (59.7%), followed by diabetic nephropathy (17.0%) and hypertension (9.1%). The mean patient body mass index was 22±3 kg/m^2^. At the beginning of the study, 141 (55.7%) of the patients were taking active vitamin D, and 182 (71.9%) of the patients were taking a phosphate binder medication. None of the patients were receiving magnesium carbonate treatment. One hundred and ninety-two (75.9%) patients were taking an angiotensin converting enzyme inhibitor or an angiotensin receptor blocker.

In the first year, 14 patients died, and 5 patients were transferred to hemodialysis. The follow-up time in 24 patients was less than one year. 15 patients did not have serum magnesium level in the first year. As mentioned above, 13 patients in previous hypomagnesemia group and 45 patients in normal magnesium group were not included. Eventually, 195 subjects were enrolled in the analysis of the changing of magnesium.

### Magnesium levels

**Fig 2:**
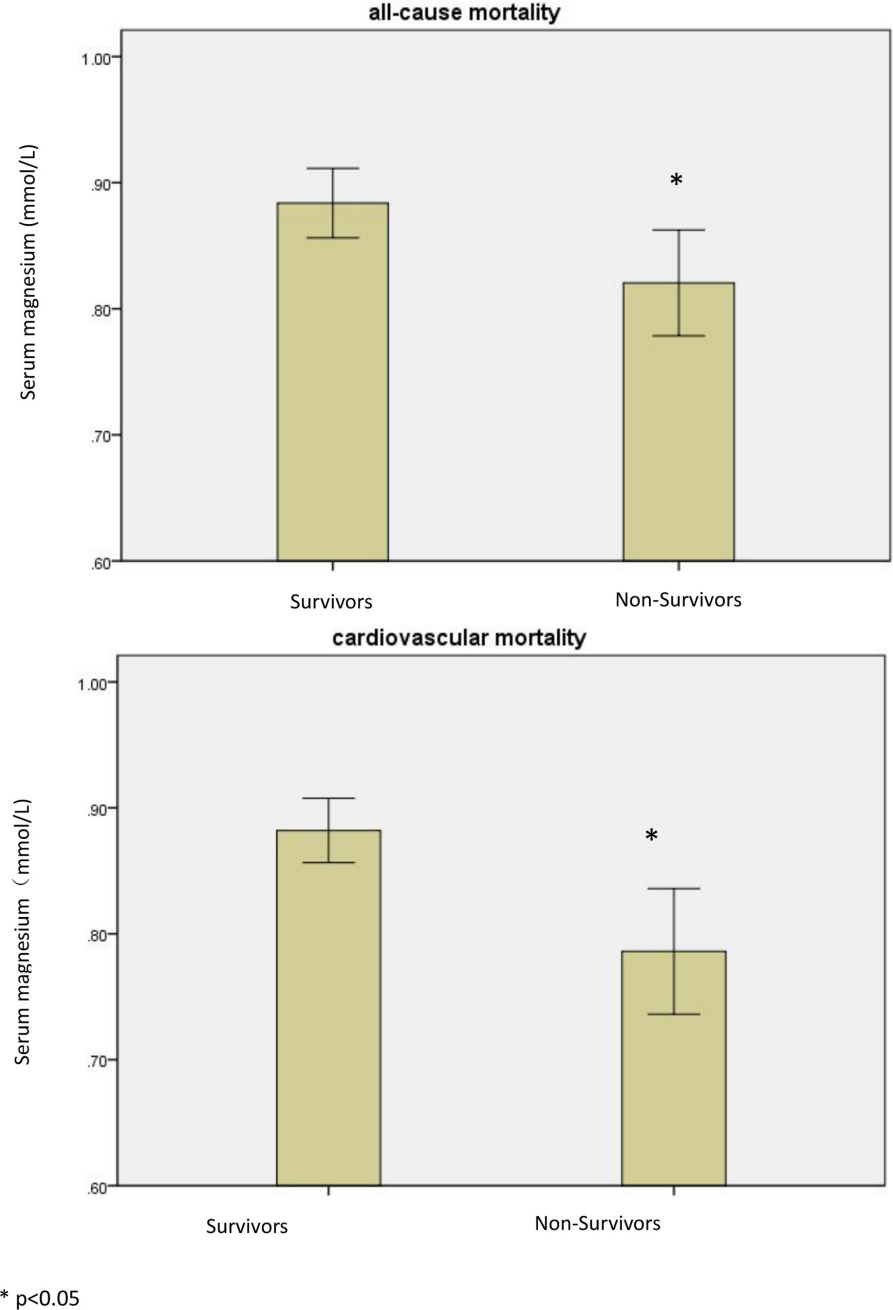
Patients who died from either all-cause or cardiovascular-only cause had significantly lower magnesium levels.

The baseline characteristics of patients stratified by serum magnesium levels are presented in Table 1 and S1 Table. The patients were divided into two groups according to the level of serum magnesium, hypomagnesemia (<0.7 mmol/L) and normal magnesium (≥0.7 mmol/L). Because relatively few patients (n = 11) presented with hypermagnesemia (serum ≥1.2 mmol/L), we included these patients in the normal magnesium group. A total of 36 patients (14.2%) suffered from low magnesium levels. Compared with the patients who had normal magnesium levels, patients with low magnesium levels had significantly lower serum hemoglobin, albumin, and calcium levels. Also, there were more men than women in the low magnesium group. We observed no significant differences between the groups in regards to age, primary renal disease, diabetes mellitus, body mass index, mean arterial pressure (MAP), ultrafiltration volume, urine output, total cholesterol, triglycerides, sodium, potassium, phosphorus, parathyroid hormone, residual renal function, or creatinine clearance. Patients who died from overall and cardiovascular causes had significantly lower magnesium levels (Fig 2). After one-year follow up, the level of magnesium increased in 19 patients of previous hypomagnesemia group. △Mg in previous hypomagnesemia group was (0.14±0.13)mmol/L. △Mg in normal magnesemia group was (-0.03±0.20)mmol/L. In all patients, serum magnesium slightly decreased, from (0.88±0.19)mmol/L to (0.87±0.17)mmol/L.

**Table 1:** Baseline demographic and clinical characteristics of PD patients stratified by baseline serum magnesium level (t/U/x^2^: different variables were compared using different methods: t-value for normally distributed variables, U-value for non-normally distributed variable, x^2^ for categorical variables).

Variables	Baseline level of serum magnesium (mmol/L)			
	<0.7	≥0.7	t/U/x^2^	p value
No. of patients (%)	36 (14.2)	217 (85.8)		
Age (year)	60±14	57±17	1.074	0.284
Gender (male, %)	27 (75.0)	113 (52.1)	6.567	0.017*
Body mass index (kg/m^2^)	22±3	22±3	0.865	0.388
Peritoneal dialysis duration (months)			-1.682	0.092
Median	19	30		
Interquartile range	9.0–46.8	17.0–51.5		
Diabetes (%)	7 (19.4)	51 (23.5)	0.288	0.592
Etiology of ESRD			4.424	0.219
Chronic glomerular nephritis (%)	20 (55.6)	131 (60.4)		
Diabetic nephropathy (%)	4 (11.1)	39 (18.0)		
Hypertension (%)	3 (8.3)	20 (9.2)		
Others	9 (24.0)	27 (12.4)		
Medication use				
ACEI/ARB (%)	28 (77.8)	164 (75.6)	0.082	0.775
Calcium carbonate (%)	26 (72.2)	156 (71.9)	0.002	0.967
Vitamin D (%)	17 (47.2)	124 (57.1)	1.232	0.267
MAP (mmHg)	96±14	96±12	-0.037	0.970
Net UF (ml/day)			-0.077	0.938
Median	123	130		
Interquartile range	-6.3–407.5	0–350.0		
Urine output (ml/day)			-1.793	0.073
Median	850	1100		
Interquartile range	700–1275	775–1425		
Hemoglobin (g/L)	87.83±18.23	98.41±18.49	-3.183	0.002*
Albumin (g/L)	25.96±6.34	29.31±5.21	-3.448	0.001*
Total cholesterol	4.70±1.03	4.72±1.13	-0.090	0.929
Triglycerides			-1.589	0.112
Median	1.36	1.30		
Interquartile range	1.02–2.11	0.95–1.87		
Sodium (mmol/L)	139.50±4.06	139.90±3.86	-0.574	0.567
Potassium (mmol/L)	3.99±0.79	4.18±0.79	-1.338	0.182
Magnesium (mmol/L)	0.64±0.05	0.90±0.17	-8.769	<0.001*
Calcium (mmol/L)	1.84±0.27	2.00±0.22	-3.743	<0.001*
Phosphorus (mmol/L)			-1.659	0.097
Median	1.25	1.38		
Interquartile range	1.09–1.47	1.13–1.66		
Intact parathyroid hormone (pg/ml)			-0.044	0.965
Median	216.30	224.00		
Interquartile range	125.65–347.62	111.30–372.15		
rGFR (ml/min/1.73m^2^)			-0.189	0.850
Median	6.47	6.73		
Interquartile range	5.66–7.94	5.21–8.98		
Creatinine clearance (L/w/1.73m^2^)			—1.589	0.112
Median	65.19	72.92		
Interquartile range	54.95–86.42	58.88–93.76		

ESRD- end stage renal disease; ACEI- angiotensin converting enzyme inhibitor; ARB- angiotensin receptor blocker; MAP- mean arterial pressure; rGFR- residual glomerular filtration rate; UF- ultrafiltation

*p<0.05.

Table 2 shows the correlations between the baseline level of magnesium and other variables. The bivariate correlation analysis showed that serum magnesium was positively associated with duration of PD (r = 0.303, p < 0.001), serum albumin (r = 0.220, p < 0.001), serum triglycerides (r = 0.160, p = 0.011), serum potassium (r = 0.156, p = 0.013), serum calcium(r = 0.299, p < 0.001) and serum phosphate (r = 0.191, p = 0.002). Serum magnesium was also negatively associated with sodium (r = -0.125, p = 0.048). No significant association was found with age, body mass index, MAP, ultrafiltration volume, urine output, hemoglobin, total cholesterol, calcium, parathyroid hormone and residual renal function, or creatinine clearance.

**Table 2:** Correlation analysis for variables and baseline serum magnesium concentrations.

Variables	*r*	p-value
Age (years)	-0.118	0.061
Body mass index (kg/m^2^)	-0.049	0.436
Peritoneal dialysis duration (months)	0.303	<0.001*
MAP (mmHg)	0.015	0.812
Net UF (ml/day)	0.103	0.101
Urine output (ml/day)	0.059	0.346
Hemoglobin (g/L)	0.106	0.093
Albumin (g/L)	0.220	<0.001*
Total cholesterol (mmol/L)	0.075	0.235
Triglycerides (mmol/L)	0.160	0.011*
Sodium (mmol/L)	-0.125	0.048*
Potassium (mmol/L)	0.156	0.013*
Calcium (mmol/L)	0.299	<0.001*
Phosphorus (mmol/L)	0.191	0.002*
Intact parathyroid hormone (pg/ml)	0.114	0.509
rGFR (ml/min/1.73m^2^)	-0.099	0.117
creatinine clearance (L/w/1.73m^2^)	-0.028	0.657

MAP- mean arterial blood pressure; UF- ultrafiltration; rGFR- residual glomerular filtration rate

*p<0.05.

### Serum magnesium and mortality

During a median follow-up period of 29 months (range: 4–120 months), 60 patients (23.7%) died, and 35 (58.3%) of these deaths were attributed to cardiovascular causes. Of the 35 cardiovascular deaths, 27 were cardiac related, 7 were due to cerebrovascular disease, and 1 was due to a pulmonary aortic embolism. Patients who died from cardiovascular causes had significantly lower magnesium levels (p = 0.005) than those who survived, as did patients who died from overall causes (p = 0.023).

**Fig 3:**
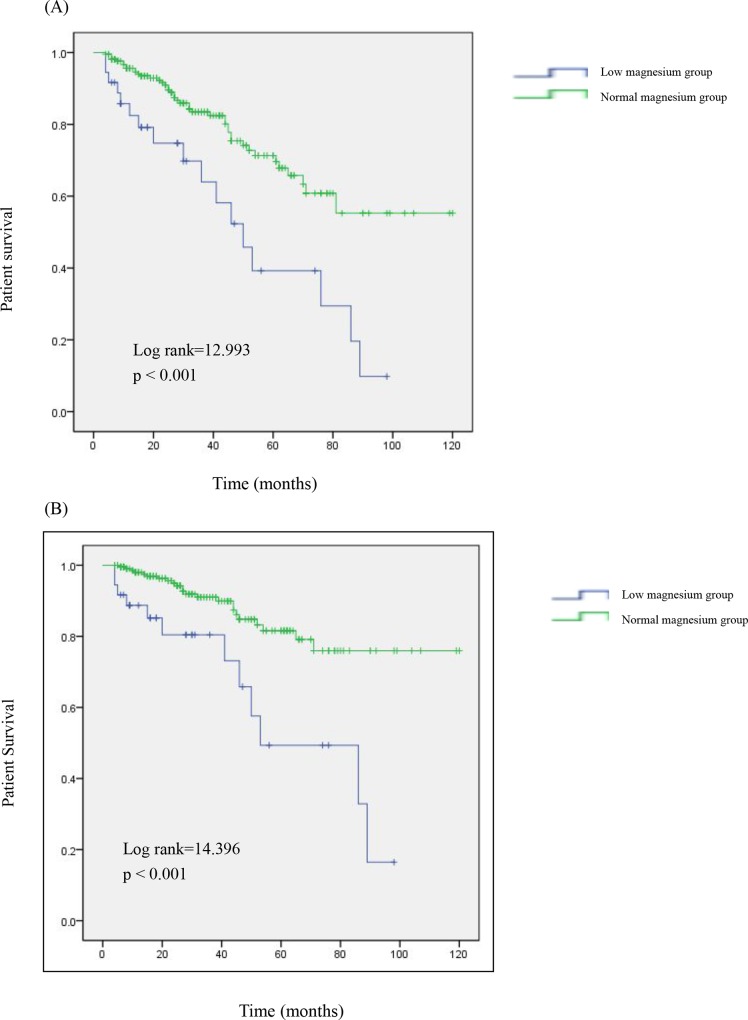
Survival curves for patients with different serum magnesium levels. Cumulative survival curves for (A) all-cause mortality and (B) cardiovascular mortality according to the category of baseline serum magnesium.

Kaplan–Meier survival curves for serum magnesium and all-cause and cardiovascular mortality are shown in Fig 3. At the end of 1, 3, and 5 years, the all-cause mortality rates were 17.5%, 36.0% and 60.8%, respectively, in the low magnesium group and 4.4%, 16.6%, and 28.7%, respectively, in the normal magnesium group (Fig 3A). Compared with that in the normal magnesium group, the survival rate was significantly lower in the low magnesium group (p = 0.04). It was the similar in male or female patients(Log Rank = 7.872, p = 0.005; log rank = 5.775, P = 0.016, respectively). The cardiovascular mortality rates after 1, 3, and 5 years were 11.3%, 19.6%, and 50.6%, respectively, in the low magnesium group and 2.0%, 9.0%, and 18.4%, respectively, in the normal magnesium group (Fig 3B). Similarly, patients in the low magnesium group had a lower cardiovascular survival rate than those in the normal magnesium group (p = 0.013).The similar results were seen in male or female patients(Log Rank = 12.364, p<0.001; log rank = 5.403, P = 0.020, respectively).

The results of univariate and multivariate Cox analysis were seen in Table 3. By univariate Cox analysis, serum magnesium was an independent negative predictor of all-cause mortality (HR = 0.041, p < 0.001) and cardiovascular mortality (HR = 0.007, p < 0.001). In sub-analysis, serum magnesium was also an independent predictor of all-cause mortality (HR = 0.044, p = 0.014;HR = 0.030, p = 0.007;respectively) and cardiovascular mortality in male or female patients(HR = 0.000, p = 0.004;HR = 0.009, p = 0.003;respectively). In the multivariate Cox proportional hazards regression analysis, we included all the significant variables from the univariate analysis as well as variables considered to be clinically relevant. This analysis also showed that serum magnesium was an independent negative predictor of all-cause mortality (HR = 0.075, p = 0.011) and cardiovascular mortality (HR = 0.003, p<0.001). In female patients, serum magnesium was an independent negative predictor of all-cause mortality (HR = 0.040, p = 0.048) and cardiovascular mortality (HR = 0.004, p = 0.007). However, in male patients, serum magnesium was not an independent negative predictor of mortality, but approaching to the significant statistics(HR = 0.052, p = 0.076;HR = 0.000, p = 0.050). However, in univariate and multivariate Cox analysis, △Mg was not an independent predictor of all-cause mortality and cardiovascular mortality.

**Table 3:** Univariate and multivariate adjusted hazard ratios for all-cause and cardiovascular mortality in patients.

	All patients						Male patients						Female patients					
	All-cause			CVD			All-cause			CVD			All-cause			CVD		
	HR	95% CI	p	HR	95% CI	p	HR	95% CI	p	HR	95% CI	p	HR	95% CI	p	HR	95% CI	p
Unadjusted	0.041	0.007, 0.233	< 0.001	0.007	0.001, 0.081	< 0.001	0.044	0.004, 0.527	0.014	0	0.000, 0.106	0.004	0.03	0.002, 0.393	0.007	0.009	0.000, 0.191	0.003
Model 1	0.075	0.014, 0.421	0.003	0.01	0.001, 0.131	< 0.001	0.066	0.006, 0.759	0.029	0.001	0.000, 0.132	0.001	0.083	0.007, 0.980	0.048	0.033	0.002, 0.657	0.026
Model 2	0.058	0.010, 0.338	0.002	0.013	0.001, 0.155	< 0.001	0.016	0.001, 0.272	0.004	0	0.000, 0.045	0.004	0.094	0.008, 1.164	0.066	0.048	0.002, 0.994	0.05
Model 3	0.075	0.010, 0.552	0.011	0.003	0.000, 0.055	< 0.001	0.052	0.002, 1.363	0.076	0	0.000, 1.013	0.05	0.04	0.002, 0.968	0.048	0.004	0.000, 0.216	0.007

Abbreviations: CVD- cardiovascular disease, HR- hazard ratio, CI- confidence interval

Model 1 was adjusted for age and gender. Model 2 was adjusted for Model 1+ DM, MAP, urine output, Net UF, rGFR, creatinine clearance and sodium. Model 3 was adjusted for Model 2+malnutrition (body mass index, serum albumin, hemoglobin level and TC, TG) and mineral and bone disorder-related factors (levels of serum calcium, phosphate, intact parathyroid hormone, prescription of Calcium carbonate, active vitamin D analogue).

## Discussion

The goal of this study was to explore the relationship between serum magnesium levels and mortality in patients undergoing PD. The results showed that serum magnesium levels were significantly positively associated with serum albumin, triglyceride, and phosphorus levels and inversely correlated with sodium concentration. In addition, a low magnesium level independently predicted cardiovascular mortality in these patients. Combined, our results revealed that hypomagnesemia has a detrimental effect on survival, as patients with low magnesium levels should be considered as at risk for all-cause and cardiovascular mortality.

In our study, 14.2% of patients suffered from hypomagnesemia. This rate is lower than the prevalences of 40.5% and 63.6% reported by Ye et al [9] and Ejaz et al [8] for PD patients, but higher than the prevalence of 8.9% reported by Cho et al [12] for PD patients. It is interesting that the prevalence of hypomagnesemia is so dramatically different among different regions, even though patients receive the same low-magnesium dialysate for PD. A future epidemiologic study with a larger sample size is warranted to more fully explore the differences among races. Meanwhile, serum magnesium level decreased slightly after one year, which was the same as the research by de Roij van Zuijdewijn et al (Δ -0.011 mmol/L/year)[13]

The pathogenesis of hypomagnesemia in the PD population is highly complex. Malnutrition may be an important cause of hypomagnesemia. Serum albumin was found to be independently associated with hypomagnesemia in our study. This finding is in accordance with other studies [9, 14]. In addition, we found that serum magnesium was positively correlated with serum phosphate, which is also consistent with other researchers [9, 15]. Phosphate intake is positively correlated with dietary protein intake [16]. Other authors have reported that magnesium depletion is closely correlated with protein-energy malnutrition in children [17],.We believe that malnutrition-related factors may be, in part, intervening factors rather than confounders for an association between magnesium deficiency and high mortality. PD patients may need to increase food intake to improve nutrition. Secondly,.patients with chronic kidney disease normally have partly depressed intestinal magnesium absorption compared with healthy individuals [18]. In the hypomagnesemia group, after one year, serum magnesium level slightly increased. We guess it maybe compensatorily alleviate in the inhibition of absorption when serum magnesium level were extremely low. It needs to be further investigated.

The cardiovascular and all-cause mortality rates were higher in the hypomagnesemia group than in the normal magnesium group, and hypomagnesemia was found to be an independent risk factor for cardiovascular mortality in PD patients, especially in female patients. This result is in accordance with the previous report on PD patients [19]. Fein et al showed that serum magnesium is a significant predictor of mortality after adjusting for age, race, sex, diabetes, and dialysis duration at enrollment. Another analysis showed that hypomagnesemia is significantly associated with an increased risk of cardiovascular mortality in Japanese HD patients [2]. Subsequently, researchers found that lower magnesium levels seem to be associated with increased CV risk markers and with higher mortality in HD patients [20]. This also holds true in chronic kidney disease patients and in the general population [21–24]. This relationship was further sustained by a meta-analysis study that found a significant inverse association between magnesium intake and/or serum levels and the risk of CVD events in different patients in 19 prospective studies including 532,979 participants [25]. However, in this study, we found that change of serum magnesium level was not an independent risk factor for all-cause mortality and cardiovascular mortality. Accounting for 10% of the difference in serum magnesium concentration, it is debatable whether this decrease is clinically relevant[13]. The relationship between time-average magnesium and mortality is warranted to be further observed.

Several mechanisms may explain the increase in CVD risk for patients with hypomagnesemia. Several cell culture and animal studies suggest a protective role of magnesium through multiple molecular mechanisms. Magnesium seems to negatively regulate vascular calcification and osteogenic differentiation through transient receptor potential melastatin (TRPM7) activity and increased expression of anti-calcification proteins [15]. Additionally, increasing magnesium levels improve cell viability and normalize the cellular release of proteins involved in vascular calcification as well as exert an anti-calcifying effect via inhibition of the Wnt/β-catenin signaling pathway [16].

The other important role of magnesium is the beneficial effect on decreasing intact parathyroid hormone[26], which is considered an independent risk factor for vascular calcification, left ventricular hypertrophy and mortality in CKD patients. A number of studies have shown that magnesium can modulate the secretion of iPTH in a similar way to calcium, through the binding to the calcium-sensing receptor (CaSR)[27] However, magnesium is 2.5-to-3-fold less potent than calcium on a molar basis in suppressing iPTH secretion[28]. Regarding the effect of magnesium on iPTH, a significant linear inverse correlation is present.But in our study there was no relationship between Mg and PTH in PD patients, which would necessitate further research on the possible role of PTH in the association between serum Mg and clinical outcome.

Malnutrition is highly prevalent in PD patients, especially in patients with hypomagnesemia [29].Our study had the same finding. Malnutrition is a one component of the malnutrition, inflammation, and atherosclerosis (MIA) syndrome, which are indicative of an increased risk of CVD and cardiovascular mortality [30]. Unfortunately, hypersensitive C reactive protein data were not available in our database; thus, the relationship between levels of this protein and Mg levels and inflammation in association with mortality cannot be determined in our patients. In this context, we believe that malnutrition may be, in part, an intervening risk factor rather than a confounder of the association between magnesium deficiency and high mortality.

In our study, serum magnesium was negatively associated with sodium, which means that patients with hypomagnesemia have hypernatremia. Elevated sodium and dehydration stimulate inflammatory signaling in endothelial cells and promote atherosclerosis, leading to CVD [31]. This may be another mechanism by which hypomagnesemia leads to high cardiovascular mortality. Our study also found that the over-all mortality in the group with hypomagnesemia was higher than that in the group with normal magnesium levels. Furthermore, hypomagnesemia was an independent predictor of all-cause mortality. A similar finding was reported by Sakaguchi et al [2], who reported that hypomagnesemia in HD patients is a significant predictor of not only cardiovascular, but also non-cardiovascular mortality, especially in infection-related deaths. The mortality rate from infection was the second highest in PD patients. Magnesium plays a key role in immunity, as both a cofactor for immunoglobulin synthesis and immune cell adhesion.

There were some limitations in the present study. This was a retrospective study with a relatively small sample size and, consequently, limited statistical power for the tests applied. Other data representing overall nutritional status such as subjective global assessment, anthropometry, and dietary protein intake were not available for the analysis. In our study, we analyzed the association between baseline serum magnesium levels and mortality, rather than serum magnesium variability with respect to mortality in PD patients. Thus, prospective studies with a larger sample size and more robust statistical analysis are required to confirm this association. And interventional studies are warranted to examine whether correction of hypomagnesemia ameliorates adverse outcomes in this population.

In conclusion, our results revealed an independent relationship between hypomagnesemia and an elevated risk of cardiovascular mortality in PD patients. These findings, along with those of previous studies in this area, suggest that clinicians could use hypomagnesemia as a risk assessment tool to identify PD patients with higher risk of mortality.

## Supporting Information

S1 Table The data of the patients with PD. (PDF)
