# Supplementary material for: Hypomagnesemia Is Associated with Increased Mortality among Peritoneal Dialysis Patients
Source: PLoS One. 2016 Mar 29;11(3):e0152488. doi: 10.1371/journal.pone.0152488 (PMC4811555; doi:10.1371/journal.pone.0152488)
Supplement: S1 Table — (PDF) [file pone.0152488.s001.pdf]

follow-up time	susvive	CVD	age	gender	BMI	etiology	DM	MAP	Hb	Alb	TC	TG	K	Na
98	0	0	33	1	18.8	7	0	73.33	76	28.4	3.3	1.09	5.11	131.3
4	1	1	71	2	17.58	1	0	93.67	54	18.6	2.91	0.63	3.02	137.2
4	1	1	86	1	18.42	1	0	78	69	11.5	4.73	1.35	2.29	129.3
4	1	0	82	1	19.84	7	0	94	113	30.5	3.86	0.92	3.73	139.6
4	0	0	83	1	19.78	4	0	86.67	96	23.1	5.42	0.76	4.33	141
5	1	1	73	1	19.49	4	0	63.33	118	13.6	4.04	1.01	3.63	132.9
5	0	0	52	1	25.06	1	1	89	67	30.3	2.06	0.62	3.77	144.6
5	0	0	36	1	15.94	1	0	104	129	36.8	7.13	1.14	6	142.8
6	0	0	39	2	17.6	1	0	106.7	97	32.2	4.76	1.98	4.07	142.1
6	0	0	44	2	24.91	6	0	100.7	71	36.1	7.32	2.17	3.03	142.2
6	0	0	45	2	18.83	1	0	117.3	70.2	30.7	4.93	1.38	5.41	140.6
6	0	0	48	1	22.86	2	0	96	115	32	4.25	1.52	4.01	146.4
6	1	0	73	2	21.64	1	0	102	104	38.6	5.42	2.54	4.68	142
6	1	1	53	2	20.96	1	0	83.33	95	15.2	3.38	1.11	5.12	138
6	0	0	73	1	25.95	1	0	93.33	109	37.5	5.04	1.52	5.15	147.4
6	1	0	70	1	27.99	3	0	83.33	118	33.1	4.04	2.32	3.84	141.1
7	0	0	64	1	20.28	2	1	101.3	90	26.6	5.94	1.51	4.72	146.7
7	0	0	50	1	18.54	2	1	96.67	117	24.4	3.51	0.38	3.59	141
7	0	0	32	1	19.15	1	0	103.3	80	31.7	5.59	1.24	4.33	140.1
7	0	0	53	1	27.34	1	0	100.7	76	32.3	3.63	1.33	3.87	147
7	0	0	68	2	21.48	1	0	98	90	37.1	4.99	1.47	3.93	145.6
7	0	0	58	2	22.96	4	0	103.3	92	27.1	5.79	8.71	2.81	139.1
7	0	0	32	1	20.76	1	0	94	118	34.9	3.54	0.99	4.15	142.8
7	0	0	59	2	20.78	2	1	90	126	36.7	4.31	0.27	4.61	148.1
8	1	1	52	1	20.31	1	0	98	69	29.1	4	0.83	4.65	141.9
8	0	0	81	2	15.4	1	0	85.33	120	24.6	5.78	1.02	3.39	143.9
8	0	0	76	1	20.86	2	1	91.67	107	29.9	4.74	0.94	4.48	140.8
8	0	0	42	2	24.65	2	1	109.3	112	27.3	8.63	1.78	3.89	143.9
8	1	1	86	2	23.44	1	0	67.33	73	24	4.82	1.27	3.34	142.6
9	0	0	48	1	19.14	1	0	113.7	106	32.9	3.93	2.05	4.45	145.7
9	0	0	56	1	24.62	1	0	96.67	92	31.6	5.5	2.82	4.8	141
9	1	0	52	1	22.15	7	1	90	87	25.2	5.75	5.71	2.92	138.8
9	0	0	54	1	21.26	1	0	100	71	24.2	4.94	3.45	4	138
9	0	0	53	2	23.23	1	1	106.7	101	37.8	4.65	2.06	3.66	143.1
10	0	0	30	1	19.03	1	0	115	53	28.3	5.06	2.47	4.9	142
10	1	0	47	2	19.63	2	1	90.67	76	18.7	7.18	5.83	4.68	140.4
10	1	1	64	2	28.35	2	1	106.7	80	27.1	4.81	0.85	6.86	137
11	1	1	72	2	19.53	2	1	101.7	94	20.2	4.92	1.96	2.94	139.1
11	0	0	56	1	23.88	2	1	99.67	133	31.8	3.23	1.13	3.05	145.2
11	0	0	74	1	19.03	3	0	100.7	90	31.9	5.28	0.72	4.08	141.9
11	1	0	76	1	17.76	3	0	80	88	25.5	4.17	0.92	5.8	136
11	0	0	80	1	21.91	3	0	88	117	32.8	4.45	1.07	4.7	140.7
11	0	0	43	2	17.19	1	0	93.33	111	29.2	5.28	1.14	4.9	140.4
11	0	0	29	2	17.77	12	0	108	101	38.1	6.13	1.87	4.49	144.3
12	1	0	76	1	23.62	1	0	102.7	62	22.6	4.66	2.39	4.19	142.6
12	0	0	60	2	18.42	1	0	95	101	37.1	5.67	1.8	4.56	143.5
12	0	0	76	2	17.22	1	0	93.67	146	29.6	5.2	1.54	4.86	139.5
12	0	0	54	2	18.53	1	0	106.7	116	34.4	6.1	3.6	3.6	143.4
13	0	0	71	1	20.76	10	0	83.33	119	31.6	4.45	1.63	3.89	143
14	0	0	85	2	20	1	0	88	110	20.8	5.24	0.66	2.6	141.3
14	1	0	53	1	23.24	3	0	88.67	74	29.2	3.83	0.69	4.26	136.8
14	1	1	83	2	24.14	3	0	76.67	121	31.4	4.95	1.47	4.4	147
15	1	1	58	2	23.31	6	0	90	86	33.4	5.67	1.56	5.12	133.7

follow-up time	susvive	CVD	age	gender	BMI	etiology	DM	MAP	Hb	Alb	TC	TG	K	Na
15	1	1	72	1	21.99	7	0	118.7	56	18.3	3.5	0.95	4.5	139.8
15	0	0	56	1	19.1	4	0	114.3	116	27.2	5.11	0.51	3.97	142.3
15	0	0	37	2	19.14	1	0	108	106	29	4.56	2.26	3.27	141.3
16	0	0	82	1	23.53	1	0	104.7	89	24.9	3.38	1.03	4.16	138.9
16	0	0	52	1	25.93	2	1	106	65	28.3	3.79	3.4	4.39	139.5
16	0	0	74	1	22.05	7	0	83.33	96	28.3	4.31	1.1	4.31	137.9
16	0	0	46	2	22.19	1	0	102.7	110	30.7	4.87	1.51	4.29	139.4
16	0	0	42	2	22.22	1	0	101	82	28.7	5.31	3.55	3.51	141.8
16	0	0	60	1	26.47	2	1	85	116	33.4	3.78	1.5	4.98	142
16	1	0	44	2	21.3	1	0	93.33	113	33.5	3.63	1.56	5.31	139.1
16	0	0	47	2	20.55	1	0	111	92	28.7	3.41	1.42	4.05	140.5
16	0	0	45	2	21.88	1	0	95.67	86	29.5	4.87	0.87	3.54	142.4
16	0	0	21	1	19.16	1	0	88	120	31.9	4.83	1	4.37	137.6
16	0	0	80	1	19.84	2	0	88.67	128	33.6	3.26	1.33	4.45	140.4
17	0	0	52	1	20.83	7	0	100.7	95	25.3	4.2	0.78	3.31	142.2
17	0	0	62	1	22.77	1	1	102.3	83	28.7	5.28	1.65	4.99	138.5
17	0	0	56	2	19.78	1	0	110	93	26.1	4.6	1.46	3.69	139
17	0	0	58	1	23.23	1	0	96.67	85	27.1	4.5	0.82	4.23	138.3
17	0	0	64	1	22.49	3	0	76.67	113	25.4	3.92	4.76	3.58	143.5
18	0	0	67	1	21.11	7	0	116.7	100	24.4	5.61	1.12	3.2	143.2
18	0	0	68	1	28.03	2	1	87.33	91	28	3	1.25	3.32	143.1
18	0	0	44	1	29.41	1	0	110	114	33.5	6.14	2.56	4.06	142.5
18	0	0	45	1	22.49	1	0	96.67	98	30.1	4.61	2.77	3.28	124.7
18	0	0	69	2	31.25	6	0	113.7	124	27	5.5	3.61	3.45	140.8
18	0	0	72	1	25.4	1	0	86.67	141	36.3	6.74	2.73	4.67	141.5
18	0	0	31	1	22.04	1	0	106.7	106	38.4	6.83	2.24	5.36	136.8
19	0	0	64	1	20.76	1	1	105.3	71	28.8	3.81	0.8	4.56	152.6
19	0	0	70	1	22.84	1	0	98.67	86	27.5	3.79	0.79	5.21	139.9
19	1	1	82	2	21.37	2	1	95.33	131	25.7	2.85	1.47	4.27	141.7
19	0	0	51	1	23.56	3	0	110	105	31.6	5.3	5.69	3.43	140
20	1	1	38	1	22.49	1	0	92	52	21.4	3.86	1.13	4.48	141.3
20	0	0	44	2	25	1	0	106.7	97	28.9	4.63	4.44	4.65	147
20	0	0	86	2	17.78	3	0	84	101	21.1	5.14	0.82	3.42	142.8
20	0	0	61	2	20.96	4	0	94	108	37.8	6.4	3.48	4.11	146.7
20	0	0	88	1	22.31	3	1	86.67	92	30	3.73	0.65	3.87	138.9
21	0	0	46	1	21.11	1	0	118.7	144	30.2	4.72	1.54	4.8	139.9
21	0	0	40	2	18.49	1	0	96	137	30	4.66	0.97	4.61	144.8
21	0	0	30	2	23.23	1	0	90	148	40.1	7.53	2.56	4.03	139.5
22	1	1	65	2	20.03	2	1	101.7	104	21.3	4.39	0.93	4.26	133.5
22	0	0	48	2	21.34	1	0	94.67	100	33.8	5.58	1.45	4.18	142
22	0	0	68	2	25.28	3	0	96.67	101	29.4	6.13	2.06	3.71	143.7
22	0	0	50	2	18.25	1	0	101.3	111	34.5	4.78	1	5.34	145.2
22	0	0	48	2	21.91	1	0	102.3	83	29.9	2.68	0.63	4.02	138.6
23	0	0	59	1	16.07	6	0	108.3	91	26.6	3.47	0.86	3.98	143.9
23	1	0	73	1	24.22	2	1	99.33	121	30	3.72	0.83	3.77	144.8
23	0	0	87	2	23.73	1	1	90	98	28.8	3.46	1.38	4.89	141.6
24	0	0	51	1	25.61	1	0	102.7	73	26.5	4.75	1.27	5.36	144.7
24	1	1	33	2	21.48	1	0	93.67	105	25.9	4.41	1.21	3.93	136.2
24	0	0	65	2	21.64	2	1	91.67	89	33.3	5.57	2.41	4.27	144.7
24	0	0	34	2	23.44	1	0	96	95	36	4.67	1.66	5.11	137.1
25	0	0	59	1	22.03	1	1	96.67	95	31.1	3.37	1.22	5.65	141.4
25	1	1	63	2	26.17	2	1	47	84	29.2	2.92	0.98	5.29	142.8
25	0	0	36	1	20.96	1	0	130	111	32.2	6.18	0.79	4.13	136.5

follow-up time	susvive	CVD	age	gender	BMI	etiology	DM	MAP	Hb	Alb	TC	TG	K	Na
25	0	0	50	2	19.53	1	0	90.67	85	30.2	4.44	1.76	3.63	142.9
25	1	0	75	1	23.56	2	1	101.7	102	16	6.08	1.27	3.85	143.1
25	0	0	52	1	20.76	1	0	85.67	107	25.7	4.31	0.58	3.84	139.3
26	0	0	44	2	25.07	2	1	96.67	75	19.6	5.04	1.36	4.9	136
26	0	0	86	2	20.27	1	0	100	98	26.6	5.95	3.25	4.94	133.2
26	1	0	65	2	23.43	2	1	84	112	23.4	5.39	1.57	2.64	137.3
26	0	0	29	2	19.19	1	0	91.67	106	32.1	4.61	1.31	4.55	139
26	0	0	48	2	22.06	2	1	100	101	23.4	6.05	0.89	3.68	140.9
26	0	0	38	2	18	1	0	102.3	119	38	4.06	1.22	3.78	136.4
27	1	1	93	1	20.2	3	0	96.67	89	29.4	3.94	1.87	2.71	142.8
27	0	0	20	1	34.15	1	0	93.33	94	37.5	6.39	3.21	5.37	140.3
27	0	0	38	1	24.8	1	0	103.3	81	27.1	2.98	1.02	4.6	143
27	0	0	77	2	21	1	0	100	103	30	3.26	1.49	4.6	140
27	0	0	23	2	18.83	1	0	100	65	30.9	3.84	0.75	3.57	139.6
27	1	1	79	2	19.53	2	1	84	101	24.7	4.59	0.56	3.35	134.8
28	0	0	41	1	24.22	1	0	90.67	106	31.7	4.19	1.5	4.2	141
28	0	0	72	1	22.68	7	0	99.33	88	22.2	5.49	1.45	4.4	140
28	1	1	68	1	25.09	2	1	83.33	100	20.2	3.4	0.82	4.15	140.2
28	0	0	58	1	19.55	2	1	120	88	30.4	4.88	2.17	5.3	146
29	0	0	51	2	21.58	7	0	105	86	31.8	4.62	1.92	5	140
29	0	0	72	2	14.22	1	0	83.33	107	26.8	4.28	1.06	4.1	138
29	1	0	77	1	18.34	1	0	126.7	68	27.4	4.46	1.88	5.16	140.3
30	0	0	33	1	24.84	1	0	100	111	38.1	4.16	2.21	4.3	145
30	1	0	67	2	20.83	3	1	101.3	103	22	5.91	2.13	3.9	143
30	0	0	45	2	19.53	1	0	111.7	106	27.8	5.04	9	4.5	139
30	0	0	49	1	22.49	9	0	112.7	111	31.2	5.84	1.89	4.3	140
30	0	0	44	1	18.56	7	0	92	75	17.9	3.7	1.38	3.17	137
30	0	0	27	1	18.56	1	0	99	118	27.2	4.18	0.89	5	140
31	0	0	58	1	22.04	1	0	123.3	67	24	4.26	1.37	3	140
31	0	0	60	1	20.9	1	0	86.33	88	23.3	4.64	1.08	3.97	139.3
32	1	1	39	2	17.09	1	0	100	111	26.3	7.26	1.41	4.08	141.1
32	0	0	51	2	22.04	1	0	90.33	91	22.1	4.91	1.18	4.2	146
32	1	0	76	1	14.88	2	1	86.67	73	16.2	3.53	1.25	3.22	132.6
32	0	0	56	1	21.22	4	0	106.7	106	35.5	5.73	2.88	4.37	141.4
32	0	0	78	2	18.43	1	0	73.67	103	28.9	3.56	1.08	4.7	144
32	0	0	76	1	20.76	2	1	105.3	73	18.1	4.69	1.24	3.9	138
33	1	0	71	2	18.02	7	1	122	56	18.5	3.87	1.12	4.16	133.5
33	0	0	78	2	24.2	2	1	101.3	120	31.6	3.27	0.49	5.16	140.6
33	0	0	85	1	21.09	7	0	96.67	111	27	4.77	2.14	3.7	144
34	0	0	57	1	24.17	1	1	93.33	101	33.6	4.54	1.4	3.97	139.8
34	0	0	52	2	26.17	1	0	81	69	31.3	4.85	3.3	3.34	138.7
35	0	0	50	1	24.39	2	1	95.33	98	29.5	4.9	1.46	4.71	144.7
35	0	0	41	2	18.73	1	0	74.67	92	28.3	5.73	1.93	3.41	136
36	1	0	71	1	23.12	3	1	103.3	92	22.7	4.83	0.84	4	140.6
36	0	0	82	2	20.13	3	0	76.67	122	28.4	4.09	1.02	3.4	130.6
36	0	0	50	2	19.53	1	0	80	129	32	8.17	1.29	3.18	131.2
36	0	0	29	1	18.45	1	0	89.33	117	37.7	4	0.8	3.91	137.3
36	0	0	55	2	25.39	1	0	97.33	78	30.6	6.45	3.41	4.65	134.9
37	0	0	38	1	22.49	1	0	100	88	31.9	6.77	1.75	5.11	138.1
37	0	0	43	1	24.02	1	0	110	90	36.1	5.75	2.91	3.88	138
37	0	0	78	2	22.31	3	0	82.33	81	24.8	2.41	0.84	5.11	136.9
38	0	0	83	1	22.49	7	0	76.33	91	25.9	3.16	0.42	2.79	137
38	0	0	56	1	23.74	1	0	86.67	97	23.6	3.83	1.14	2.61	131.8

follow-up time	susvive	CVD	age	gender	BMI	etiology	DM	MAP	Hb	Alb	TC	TG	K	Na
38	0	0	49	1	20.87	1	0	83.33	81	25.6	7.13	5.52	4.87	136.3
38	0	0	43	1	27.68	1	0	98.67	122	38.3	4.11	0.92	3.88	139.7
39	1	1	79	1	20.31	3	1	96.67	85	26.2	4.98	1.73	3.73	140.7
39	0	0	69	1	20.48	1	0	92	95	23.9	4.44	0.71	4.04	140.4
39	0	0	45	1	27.36	2	1	113.3	99	27.1	5.34	1.3	3.23	143.2
40	0	0	48	2	20.58	1	0	93.33	105	31.6	6.96	2.03	3.58	134.8
41	1	1	67	1	21.45	1	0	95	114	21.9	5.62	0.81	4.35	139
41	0	0	50	1	15.56	7	0	80	97	26.3	4.09	1.19	2.93	137.9
42	0	0	72	2	16.37	1	0	91	126	28.6	3.66	0.7	4.36	141
42	0	0	48	1	26.99	1	0	89.33	89	26.3	4.81	1.07	4.57	137.5
42	0	0	69	1	23.18	1	0	94.33	85	23.7	4.04	1.46	3.88	137.9
42	0	0	52	2	21.37	1	0	113.3	75	34	4.36	1.04	4.78	139.4
43	0	0	66	1	24.09	1	0	84	122	28.4	4.51	0.7	4.03	138.4
43	0	0	41	1	24.69	1	0	95.33	130	30.1	4.25	0.99	4.3	141
44	0	0	29	1	18.48	1	0	96.67	103	30.1	4.02	1.37	4.29	139.5
44	1	1	75	2	15.82	1	0	103.3	75	29.9	4.81	0.7	5.37	139.6
44	1	1	81	2	22.89	2	0	93.33	97	21.3	4.51	1.82	2.14	138.6
45	1	1	69	2	28.72	3	1	93.33	96	32.3	6.49	3.45	4.84	138.9
45	1	0	82	1	19.14	1	0	90.67	104	27.7	3.16	0.59	3.58	136.9
46	1	1	76	2	18.61	7	0	95.33	55	15.9	6.01	0.93	2.58	138.2
46	1	0	74	1	20.59	1	0	88.33	134	38.6	4.52	1.47	4.41	132.5
46	0	0	63	2	26.78	1	0	98.67	94	31.4	3.53	1.54	4.1	135.6
46	1	1	92	2	24.13	2	1	96.67	87	21.9	2.93	2.74	1.97	134.5
46	0	0	61	1	21.63	1	0	93.33	88	27.9	4.01	0.58	4.43	137
46	0	0	93	1	21.45	1	0	113.3	103	32.3	4.84	1.52	4.06	138.8
47	0	0	51	1	21.16	1	0	120	71	28.2	3.51	1.31	4.91	132.1
48	0	0	56	2	24.39	1	0	83.33	85	27.1	5.06	1.24	3.79	138.1
49	0	0	62	1	26.22	1	0	86.67	84	25	4.46	2.96	3.46	141.6
50	1	1	82	2	20.28	3	0	86.67	95	32.2	4.6	1.41	4.96	138.8
50	0	0	51	2	21.06	1	0	96.67	89	28	2.86	1.23	3.32	139.1
50	1	0	80	1	23.99	1	1	90.67	96	34.3	4	0.82	3.61	139.9
51	0	0	58	1	23.53	8	0	88.67	71	26.7	3.58	0.76	4.03	141.6
51	0	0	59	2	23.23	2	1	101.3	100	31.5	4.47	3.52	3.26	138.9
51	0	0	27	1	23.03	1	0	87.33	123	32.3	5.36	2.53	5.3	140.4
52	0	0	58	1	25.01	1	0	80	82	21.9	2.09	1.08	5.25	139.2
52	0	0	74	1	17.65	2	1	126.7	76	22.6	5.93	0.78	4.21	138.5
52	1	1	86	2	23.11	3	0	84	74	22.1	3.97	0.65	2.81	136.3
53	1	1	43	1	21.8	7	0	83.33	94	17.3	5.34	0.71	3.54	140.2
54	0	0	49	2	20.81	1	0	120	89	27.6	6.7	1.69	3.61	136.3
54	1	1	53	1	21.8	2	1	100	70	33.9	4.66	1.13	5.59	147.8
54	0	0	37	2	19.53	1	0	84.67	109	38.6	5.5	8.33	3.57	139.6
55	0	0	40	2	18.43	1	0	79.67	125	37.2	4.88	1.63	3.28	135.6
55	0	0	34	1	18.25	1	0	99	98	31.6	5.79	2.05	6.04	132.4
56	0	0	64	2	27.34	2	1	90.33	105	30.5	7.58	3.01	4.8	134
57	0	0	56	2	17.78	1	0	102	105	33	5.19	2.25	3.87	139.5
58	0	0	54	1	26.3	1	0	113.3	84	26.3	4.43	0.54	3.24	140.3
60	0	0	78	1	24.84	8	1	77.67	102	36.2	3.83	1.6	4.35	143.1
60	0	0	53	2	17.31	4	0	83.33	80	31.9	4.41	1.44	4.47	143
61	1	0	56	1	22.2	2	1	96.67	93	27.7	5.18	1.2	3.62	137.8
61	0	0	50	2	19.56	2	1	107.7	117	28.4	4.16	1.13	4.93	136.9
61	0	0	67	2	23.44	2	1	74.67	93	26.1	5.44	3.71	3.92	135.5
62	0	0	49	1	20.76	4	0	86.67	93	33.8	3.37	0.78	5.52	141.4
62	1	0	62	1	26.89	2	1	97.33	82	25.6	7.21	1.28	4.33	139.7

follow-up time	susvive	CVD	age	gender	BMI	etiology	DM	MAP	Hb	Alb	TC	TG	K	Na
62	0	0	53	1	21.39	1	0	116.7	111	35	5.02	1.3	3.81	139.8
63	0	0	48	2	16.61	1	0	90	87	30.2	4.65	1.97	4.36	138.2
63	0	0	77	1	20.2	2	1	77	112	21.3	5.11	1.02	2.61	135.3
64	0	0	54	2	24.14	1	0	103.3	74	35.9	4.44	1.93	4.65	144.9
65	1	1	66	1	21.3	1	0	93.33	78	23.1	3.93	0.63	5.96	135.8
66	0	0	72	1	18.37	1	0	80.67	106	28.9	5.49	1.41	2.83	141
66	0	0	25	2	16.61	1	0	73.33	54	28.7	3.44	0.64	3.29	133.4
66	0	0	51	1	20.9	1	0	106.7	113	31.1	3.49	1.83	4.03	128.3
67	0	0	24	2	24.01	1	0	127	105	26	5.59	1.33	2.88	138
67	0	0	81	1	25.78	3	1	103.3	130	29.6	3.73	1.47	3.96	136.2
70	1	0	76	1	15.79	1	0	81.33	78	20.8	4.78	0.71	3.34	141
70	0	0	31	2	19.22	1	0	106.3	92	27.4	4.97	1.1	4.18	138.1
71	0	0	52	1	23.49	4	0	92	95	33.2	2.72	1.3	4.18	148.5
71	0	0	61	1	24.61	1	0	85.33	91	30.5	4.51	0.86	3.04	143.2
71	0	0	46	1	16.63	1	0	120	73	29.5	3.47	0.53	4.58	142.5
71	0	0	55	1	20.76	1	0	100	99	34.4	4.65	0.92	3.2	141.5
71	0	0	78	1	24.03	3	0	89	80	29.6	4.2	1.57	4.28	143.9
71	1	1	72	2	20.83	1	0	103.3	75	25.6	6.03	1.16	4.08	143.1
74	0	0	60	1	22.59	1	0	118	105	33.8	3.81	1.1	3.07	141.6
74	0	0	39	1	18.14	1	0	100	84	27.2	4.03	2.93	5.01	139.4
76	1	0	78	1	20.59	1	0	93.33	89	21.8	5.02	1.02	3.02	144.2
76	0	0	46	1	17.36	6	0	81	118	27.8	3.83	1.11	4.44	138.3
76	0	0	58	2	28.84	3	1	98.33	90	35.8	5.58	2.11	5.03	141.1
76	0	0	62	1	28.08	4	1	110	101	36.8	4.53	1.26	3.88	143.1
78	0	0	57	1	24.38	1	0	98.33	98	37.7	5.16	1.64	4.66	141.2
78	0	0	33	1	26.54	1	0	81.67	110	35.4	7.27	3.29	4.24	141.2
79	0	0	42	2	18.31	1	0	106	106	38.6	4.25	0.98	5.29	138.8
80	0	0	40	2	23.63	1	0	90	101	39.2	4.9	1.03	4.69	141.2
81	1	0	73	1	15.73	1	0	96.67	119	34.5	4.68	1.21	5.83	137.9
83	0	0	42	2	17.51	1	0	100.7	59	15.2	4.14	1.18	3.84	123.5
86	1	1	63	2	16.89	1	0	70	104	19	4.97	1.45	2.74	139.5
89	1	1	58	2	27.64	1	0	92.67	79	35.1	3.82	0.86	5.24	137.3
90	0	0	43	1	22.32	1	0	112	110	28.8	3.24	1.13	4.65	138.2
90	0	0	42	2	20.58	1	0	96.67	105	31.9	7.32	2.42	5.2	135.4
92	0	0	35	2	18.73	1	0	135	106	23.6	6.34	1.35	4.41	135.5
98	0	0	48	2	18.82	1	0	91.67	129	36.2	4.38	1.09	4.69	135.3
99	0	0	48	1	19.95	1	0	103.3	82	24.7	5.28	1.17	4.59	142.8
104	0	0	52	2	20.66	1	0	87.33	85	24.2	5.7	1.53	4.51	140.2
107	0	0	66	2	21.23	1	0	106.7	109	32	4.24	0.95	5.22	139.1
119	0	0	55	1	25.71	1	0	126	130	35.8	3.25	1.13	3.83	138.7
120	0	0	42	2	25.22	2	0	103.3	92	27.3	5.36	2.15	5.1	140.9

Ca	Mg	mg 1-year	P	PTH	urine output	r-GFR	UF	Ccr	ACE/ARB	Calcium carbonate	Vitamin D
1.22	0.69	0.69	0.85	840.9	1000	5.65	300	49.28	1	1	1
1.79	0.69		1.44	172.4	800	6.74	615	55.25	1	1	1
2.16	0.55		0.68	402	100	5.69	500	42.97	0	1	1
1.77	0.82		1.44	274.9	2000	7.86	50	68.05	1	1	1
2.16	0.91		0.84	100.7	1300	4.33	40	53.45	1	1	1
1.46	0.65		1.13	85.7	600	7.22	500	64.73	0	1	0
1.79	0.76		0.9	405.7	1200	6.45	-345	66.852	1	1	0
2.05	0.92		1.97	330.4	500	8.70	570	53.376	0	1	1
1.69	0.65		1.75	386.7	800	6.50	120	96.383	0	1	0
1.5	0.78		2.4	512.8	900	4.79	155	69.142	1	1	0
1.79	0.99		1.73	587.4	1400	6.75	-15	68.02	1	1	1
1.82	0.76		0.92	333.1	750	13.48	-140	82.093	1	1	1
2.01	0.97		1.9	575.5	1000	5.44	420	84.2	0	1	0
2.02	0.7		1.68	71.5	200	7.40	505	58.86	1	1	1
2.03	0.79		1.29	123.2	2220	8.65	10	126.088	1	1	1
2.07	0.78		0.97	92.6	700	11.78	100	140.01	1	1	0
1.5	0.6		1.42	309.8	1000	9.38	100	92.21	1	0	0
1.67	0.83		1.02	57.1	500	13.37	314	97.65	0	1	1
1.88	0.75		1.88	199.8	1100	6.11	0	59.022	1	0	0
2.05	0.96		1.41	204.8	1300	6.88	-250	76.81	0	0	0
2.06	0.88		1.13	159.6	1200	9.25	30	174.797	0	0	0
2.09	1.41		1.41	37.5	1000	7.84	50	74.15	1	1	0
2.15	0.75		1.21	81.9	1100	9.55	110	86.64	1	1	1
2.17	0.86		1.36	65	2000	24.35	-350	219.48	1	1	0
1.3	0.67		2.84	146.6	700	4.19	400	42.762	1	1	1
1.82	0.75		1.07	127.9	400	10.81	150	60.816	1	0	0
1.82	0.83		1.41	347.1	1700	10.31	250	60.71	1	1	0
1.91	0.79		0.98	221.3	900	11.65	-145	129.37	1	1	1
1.92	0.83		1.64	676.6	1000	6.70	200	88.027	0	1	1
1.51	0.67		1.27	714.2	700	6.93	-165	65.38	1	1	1
1.8	0.64		1.09	276.5	1200	5.22	125	62.45	0	0	0
1.83	0.62		1.04	142.1	900	22.14	100	109.35	1	0	0
1.95	0.68		1.15	135.4	700	7.32	415	89.95	1	0	0
2.3	0.87		1.33	95.7	700	6.79	355	86.47	1	0	0
1.76	0.8		2.46	255.4	900	2.70	250	48.8	1	1	0
1.8	0.73		1.31	78.3	1100	13.85	0	81.89	1	1	0
2.1	0.9		1.45	219.9	1200	5.76	-100	66.23	0	1	1
1.62	0.74		0.92	114.3	300	9.04	405	38.47	1	1	1
1.92	1.07		1.51	247.4	1100	5.01	170	51	0	0	1
1.98	0.81		1.46	215.6	1500	12.94	-245	101.36	1	0	0
2.01	0.96		1	207.7	2100	13.66	50	158.17	0	1	0
2.07	0.76		0.9	80.9	1200	13.03	55	80.94	1	1	1
2.08	0.87		1.65	283.9	1200	6.07	0	77.1	1	0	0
2.21	0.85		3.06	62.1	100	6.93	725	59.201	1	1	0
1.89	0.54	0.65	0.88	81.8	2000	7.86	130	31.15	1	1	1
1.91	0.83		1.05	730.4	1200	11.39	0	105.202	0	1	1
1.94	0.99	0.97	1.5	157	1000	6.21	160	38.25	1	1	1
2.18	0.78	0.82	1.24	474.9	1100	6.56	30	58.906	0	0	1
2.25	0.83	0.78	1.07	707.5	1000	8.74	100	102.12	0	0	1
1.64	0.74	0.68	0.93	441.7	700	6.58	350	82.24	1	1	0
1.75	0.79	0.95	3.07	344	400	3.44	190	33.66	1	1	0
1.75	0.82	0.66	1.07	288.5	500	7.01	195	61.81	1	1	1
2	0.69	0.71	2.67	127	1350	3.41	-350	57.91	1	1	1

Ca	Mg	mg 1-year	P	PTH	urine output	r-GFR	UF	Ccr	ACE/ARB	Calcium carbonate	Vitamin D
1.78	0.78		1.5	349.4	1400	3.56	700	54.72	1	1	1
1.83	0.83	1.06	1.23	133	1300	5.27	-210	66.28	1	1	0
2.06	0.85	0.78	1.82	235.7	1000	7.21	150	84.11	0	0	1
1.3	0.54	0.61	1.64	214.5	1000	6.33	-105	63.46	1	1	0
1.82	0.58	1.04	1.66	457.6	700	9.66	410	102.26	1	1	0
1.92	0.61		1.21	55.7	1500	14.05	-145	142.53	0	1	0
1.68	0.71	0.91	1.19	435.4	1000	6.50	10	86.21	1	1	0
1.7	0.75	0.81	2.31	802.5	900	4.53	-95	69.11	0	1	1
1.75	0.88	0.94	1.18	382.6	1200	7.58	330	86.95	1	1	1
1.78	1.04	1.09	1.46	458	1800	5.10	200	66.099	1	1	1
1.82	0.86	1.09	1.83	476.2	1000	5.35	-100	57.83	1	1	0
1.92	0.84	0.83	1.69	428.8	1100	6.72	-20	96.08	0	1	1
2.1	0.81	0.71	1.66	111.6	1300	8.82	-200	111.88	0	1	0
2.13	0.74	0.81	0.99	45.3	1400	15.00	25	50.49	1	0	0
1.86	0.77	0.53	1.32	156.7	400	8.68	220	87.714	1	1	0
1.88	0.88	1.09	1.11	260.1	1100	5.44	50	61.61	1	1	0
1.9	0.72	0.87	0.89	93	800	4.70	-5	48.55	1	1	1
1.91	0.77	0.74	2.07	225.7	500	4.81	490	70.15	1	1	0
2.6	1.12	0.8	1.34	33.2	150	3.61	700	59.86	1	1	0
1.94	0.63	0.69	0.95	105.2	450	6.30	160	59.633	1	1	0
2.01	0.68	0.99	1.11	218.1	800	8.34	50	74.07	1	0	0
2.13	0.69	0.64	1.45	165.2	1000	5.47	200	65	1	0	0
1.97	1.02	0.92	2.25	396.6	900	4.46	560	30.05	1	0	1
2.05	0.78	0.82	1.11	104.6	400	3.21	550	62.39	1	1	0
2.1	0.9	1.08	1.03	130.5	1200	6.71	-224	98.81	1	0	0
2.12	1.25	1.19	1.82	335.6	2330	5.73	-400	62.06	0	1	0
1.78	0.81	0.91	1.23	189.1	1000	4.81	525	59.493	1	1	0
1.85	0.91	0.52	1.98	226.2	800	6.60	200	67.52	1	1	0
2.01	0.9	0.73	1.17	86.8	750	7.67	-135	40.89	1	0	1
2.09	0.75	0.64	1.88	394	500	3.96	380	62.27	1	1	0
1.62	0.68	0.78	2.41	423	750	2.61	435	39.342	1	1	1
1.78	0.78	0.84	1.82	609.9	2000	3.65	180	77.003	1	0	1
1.9	0.71	0.71	0.78	28.2	700	7.28	295	65.111	1	1	0
2.19	0.74	0.82	1.21	239	2100	10.06	0	131.89	1	0	1
2.36	0.87	0.69	0.89	8.1	900	7.23	-84	63.68	1	1	1
1.83	0.92	0.83	1.35	152.3	1500	8.72	15	100.13	1	1	1
1.92	0.71	0.6	1.27	605.1	600	0.30	-150	40.51	1	1	1
1.92	0.72	0.76	0.95	103.3	1900	14.33	-340	349.89	1	1	1
1.96	1.02	0.79	1.38	246	1200	13.33	75	70.49	0	1	0
1.98	0.87	0.81	1.24	551.3	1200	11.56	65	143.93	0	1	0
2.07	0.72	0.77	1.24	586.3	1500	6.31	-300	85.07	0	1	1
2.07	0.9	0.86	1.77	183.1	2000	6.37	40	101.91	1	1	1
2.17	0.98	1	1.02	111.4	900	5.46	0	57.06	1	1	1
1.78	0.75	0.66	1.23	511.2	1600	8.62	-265	94.89	0	0	1
1.8	0.89	0.89	1.04	423.5	1600	11.00	700	90.39	1	1	1
1.92	0.71	0.7	1.28	231.5	1550	6.61	-70	81.87	1	1	1
1.91	0.83	0.9	1.49	349.9	1000	3.31	30	51.888	1	1	0
2.02	0.75	0.61	1.37	234.9	1550	3.97	70	79.08	1	1	0
2.1	0.89	0.81	1.53	498.5	1350	11.33	300	42.07	1	0	0
2.21	0.9	#N/A	1.76	110.6	1250	7.63	-30	97.97	1	1	0
1.74	0.74	0.86	1.34	449	2000	7.29	10	86.4	0	2	0
1.85	0.79	0.7	0.77	278.2	1500	6.96	130	65.53	1	1	1
1.87	0.75	0.75	2.24	496	300	3.77	500	55	1	1	1

Ca	Mg	mg 1-year	P	PTH	urine output	r-GFR	UF	Ccr	ACE/ARB	Calcium carbonate	Vitamin D
1.89	0.93	1.05	1.66	293.5	1200	5.56	50	85.83	0	1	0
1.92	0.96	0.81	1	4	1200	19.75	800	110.519	1	0	0
2.16	0.78	0.75	1.13	82.3	900	6.50	-25	97.722	1	0	0
1.95	1.25	0.92	1.25	165.1	1400	7.25	30	66.628	1	0	0
2	1.24	0.52	1.65	152.4	800	5.46	60	43.66	1	1	0
2.02	1.29	1.03	1.09	225.1	1500	9.06	500	166.647	0	0	0
2.03	0.92	0.82	1.99	267.2	1500	4.39	-50	50.86	1	1	1
2.18	0.89	0.77	1.41	42.8	1100	5.48	480	77.08	1	1	0
2.79	0.85	0.64	1.28	16.3	1200	4.91	-90	100.36	0	0	0
1.7	1	0.95	2.32	767.6	250	5.49	390	45.455	0	1	0
1.86	1	0.92	2.09	469.9	2300	6.18	540	117.12	1	1	1
1.89	0.73	0.67	1.89	460.3	1000	12.43	350	75.54	1	1	1
1.9	0.83	0.65	0.54	217.5	2100	6.48	35	67.5	1	1	1
2.12	0.86		2.24	206.7	700	7.36	480	57.469	1	0	1
2.19	0.87	0.69	1.35	48.9	800	6.27	190	46.545	1	1	1
2.03	0.63	0.79	1.48	103.9	1800	12.36	-250	40.31	1	0	1
2.03	0.68	0.78	1.48	103.5	1000	7.68	-90	103.1	1	1	0
1.78	0.78	0.69	1.06	97	650	8.21	200	51.363	1	1	0
1.83	0.82	0.94	1.94	376.4	1350	9.90	0	107.66	0	0	0
1.67	0.86	0.71	2.25	400.8	1100	5.61	100	75.95	0	1	0
1.81	0.92	0.81	0.7	139.1	1350	7.45	125	84.65	0	1	0
1.91	0.79		0.98	95.1	1200	13.23	50	104.29	1	1	0
1.72	0.59	0.66	1.32	219.1	1600	7.47	175	102.44	1	1	1
2.09	0.68	0.99	1.2	208	800	6.09	80	75.84	1	1	1
1.57	0.99	1.04	2.33	345.4	800	3.59	250	51.17	1	1	0
1.84	0.8	0.87	1.11	286.4	1850	8.01	236	87.95	1	1	1
1.94	0.7	0.75	1.44	252.5	800	5.51	300	58.734	1	0	1
1.97	0.76	0.86	1.19	147.4	1400	7.12	-130	57.41	1	1	1
1.72	0.64	0.87	1.36	352	1300	5.27	35	70.456	1	1	0
2.01	0.75	0.82	1.08	122.9	1100	9.54	-200	75.07	1	1	1
1.62	0.78	0.96	2.27	422	400	7.16	-320	76.53	1	1	1
1.71	0.89	0.96	0.84	418.8	300	6.95	85	66.24	0	1	1
1.89	1		0.29	43.9	150	22.65	330	68.68	1	0	0
2.03	1.08	0.91	1.51	371.7	500	7.52	350	74.423	0	1	0
2.06	0.96	0.69	0.79	280.5	1300	6.46	-330	68.89	1	1	0
2.09	0.74	0.89	0.74	147.3	1200	24.70	150	146.09	0	0	0
1.77	0.86	0.9	1.42	249.8	500	4.83	415	66.645	1	0	0
1.96	1.29	0.95	1.35	248.6	1050	4.27	-90	49.46	1	1	1
2.27	0.81	0.82	0.98	66.1	1200	10.47	-80	80.25	1	1	1
1.92	0.87		1.09	229	1200	9.38	100	87.66	1	1	0
2.34	0.74	0.81	2.06	111.2	150	3.36	1345	38.47	1	1	1
1.92	1.04	0.81	1.57	687.3	800	6.01	300	55.15	1	1	0
1.96	0.87	0.95	2.27	183.2	1300	4.10	640	45.76	0	0	0
2.01	0.69	0.7	0.9	125.2	900	6.59	500	72.667	1	1	0
1.76	1.04	0.84	0.86	191	900	5.41	-90	83.25	1	1	1
1.87	0.79	0.9	1.95	730.8	700	5.46	315	83.89	0	1	0
2.28	1.16	1	1.22	164.8	1150	8.32	475	88.5	1	1	1
2.35	0.78	0.81	2.32	623.8	1400	5.73	-190	67.44	1	1	1
1.78	2.28	0.8	2.28	612.3	800	4.36	50	59.23	0	1	0
2.19	1	1.27	1.48	520	500	5.12	200	47.637	1	0	0
2.33	1.09		1.4	54.2	1050	5.59	1120	64.63	1	1	1
1.71	0.73	0.69	1.56	39.9	1600	4.73	-5	64.79	1	1	1
1.85	0.71	0.81	1	182.1	700	7.84	480	71.21	1	1	1

Ca	Mg	mg 1-year	P	PTH	urine output	r-GFR	UF	Ccr	ACE/ARB	Calcium carbonate	Vitamin D
2.03	0.73	0.69	1.78	112.1	1450	6.19	-112	87.36	1	1	1
2.18	1	0.83	1.57	139.9	600	5.23	125	60.07	0	1	0
1.95	0.78	0.82	1.01	152.3	400	8.93	180	52.61	1	0	0
2.08	0.93	0.85	1.76	526.1	1100	8.26	450	92.068	1	1	0
2.12	1.03	1.03	1.28	282.3	390	5.19	980	55.15	1	1	1
2.26	1.02	0.95	2.11	200.3	1300	6.21	420	105.61	0	1	0
2.02	0.54		1.27	65.1	1850	5.09	245	54.851	0	0	0
2.29	0.93	0.82	1.61	105.2	1100	13.13	-125	61.9	1	1	1
1.81	1.05	0.75	1.09	94.7	650	5.14	-90	49.687	1	1	1
1.89	0.78	0.84	1.57	311.3	1200	4.83	410	66.42	1	1	1
2.06	0.81	0.93	1.51	177.5	1800	14.07	75	142.05	1	0	0
2.2	0.87	0.82	1.02	279	1000	4.07	200	48.32	1	0	1
1.75	0.84	0.64	1.58	224	1300	5.03	150	48.08	1	1	1
2.01	0.84	0.84	1.18	93.5	800	15.67	350	58.54	1	0	0
1.86	0.92	0.88	1.86	326	3000	12.06	-250	144.11	1	1	1
1.87	1.24		1.12	372.6	1200	10.21	210	72.92	1	0	1
1.88	0.82	0.68	1.38	296.4	1150	6.05	-55	77.42	1	0	0
2.2	0.98	0.82	2.13	73	600	4.70	1200	65.16	1	1	1
2.21	1.17	1.05	1.17	119.2	1300	9.49	-400	109.36	1	1	1
1.73	0.65	0.65	1.2	323.7	500	6.24	200	65.91	1	0	0
1.89	1	1.1	0.83	263.2	800	10.25	200	69.39	1	1	1
1.92	0.87	0.96	1.35	227.2	1500	6.80	-10	91.67	1	1	1
1.94	0.85	0.78	1.14	105.4	1200	7.04	400	493.898	0	0	0
2	0.85	0.84	1.67	300	2000	7.64	-205	96.69	1	1	1
2.16	0.71	0.69	1.19	103.9	1500	10.08	0	100.56	0	1	1
1.78	0.69	0.91	1.37	334.5	1100	8.01	100	61.056	1	1	1
1.66	0.75	1.01	1.36	449.2	1100	6.98	355	104.1	1	1	1
2.2	1.12	0.97	1.65	143	700	5.41	480	75.29	0	0	0
2.29	0.69	0.9	1.45	140.2	550	16.53	100	107.58	1	1	1
1.74	1.02	1.28	2.72	445.4	750	2.15	1000	50.535	0	0	1
2.19	0.91	1.11	1.26	102.2	1200	12.67	400	131.489	1	1	0
1.86	0.96	0.96	1.54	252.8	1400	5.80	300	93.398	0	0	1
2.23	1.05	0.59	1.75	216.6	900	7.97	240	143.073	0	0	1
2.33	0.96	0.97	1.38	42.8	2000	18.17	-615	211.28	1	1	1
1.98	0.84	0.96	1.51	301.1	1625	6.49	-10	86.12	1	1	1
1.98	0.91	0.87	1.3	133.8	1000	22.23	200	234.487	1	0	0
2.04	0.81		1.12	206.8	600	5.56	110	50.05	1	1	1
1.69	0.65		1.08	293.6	500	6.45	500	56.015	1	1	0
1.52	1.03	0.92	2.22	830.5	800	3.57	250	52.8	0	0	1
2.26	1.05	1.08	1.85	90.7	200	6.87	0	74.53	1	1	1
2.55	1.7	1.7	1.97	15.3	700	4.85	200	46.835	0	0	0
2.18	1.08	0.96	0.83	312.2	1650	10.05	-200	97.88	1	1	1
3.42	1.32	0.76	1.27	19	1650	6.02	250	55.54	1	1	1
1.97	0.68	0.68	1.55	298.8	700	4.87	445	50.28	1	1	1
2.1	0.91	0.8	1.15	132.8	1250	13.29	0	68.736	0	0	0
2.34	0.77	0.87	1.3	118.5	1350	4.67	540	70.44	1	1	1
2.14	0.96	0.98	0.96	83.9	1400	12.42	315	113.7	1	1	1
2.19	0.86	1.16	1.49	245	700	4.38	480	67.202	0	0	1
2.05	0.77	0.9	1	22.6	700	8.39	610	59.042	1	1	0
2.1	0.99	0.95	1.41	87.5	3100	6.34	-375	106.92	1	1	1
2.3	0.89	1.02	0.98	4	2150	11.74	85	92.27	1	1	1
2.08	1.19	#N/A	3.69	502.7	2000	3.89	450	73	1	0	1
2.13	1.02	0.97	1.53	271.9	2600	7.64	-50	106.78	1	1	1

Ca	Mg	mg 1-year	P	PTH	urine output	r-GFR	UF	Ccr	ACE/ARB	Calcium carbonate	Vitamin D
2.23	0.81	0.9	1.4	322.7	2500	8.81	-530	103.92	1	1	1
1.91	0.96	1.07	1.55	473.2	1500	7.34	220	72.64	1	0	1
2.26	0.73	0.93	1.18	22.7	1100	12.59	256	128.11	1	1	1
2.22	0.95	0.66	1.71	86.9	1160	4.42	-400	74.16	1	1	1
1.84	0.75	0.93	1.45	339	1500	5.08	0	61.925	0	0	0
2.02	1.03	0.84	1.12	128.8	600	11.79	305	94.88	0	0	0
2.05	0.95	1.02	1.64	168.1	500	8.08	200	87.52	0	1	1
2.27	1.02	1.02	1.22	62.8	1500	6.81	300	51.078	0	0	0
2.09	0.86		1.68	140.4	825	6.54	410	60.53	1	1	0
2.15	1.17	1.06	1.46	356	900	7.34	450	94.86	1	1	1
1.97	1	0.71	1.46	177.5	400	6.72	600	56.46	1	1	0
2.29	0.84	1.09	1.89	44.4	1000	7.16	0	51.41	1	1	1
1.64	0.8	0.82	1.44	345.5	1500	13.57	0	166	1	1	1
2.06	0.92	1.21	1.34	265.5	1550	6.10	200	74.63	1	1	1
2.06	1.02	1.27	1.62	202.1	500	5.69	500	41.17	1	0	1
2.18	0.97	0.99	1.35	195.5	1500	13.52	635	139.968	1	0	0
2.18	1.03	0.91	1.24	125.3	1500	8.53	65	73.16	1	1	1
2.3	0.76	0.91	1.33	145	950	4.65	600	74.13	1	1	1
2	0.65	0.88	1.23	556.1	2000	7.96	-300	67.15	1	1	1
2	1.02	1.11	1.59	610.4	700	4.65	350	49.905	1	1	0
2.25	0.65	0.85	1.24	13.4	400	6.25	-265	52.039	0	0	0
2.03	0.83	0.64	1.24	594.3	1500	6.73	100	59.11	1	1	1
2.22	1.08	1.82	2	673.2	1200	8.48	250	84.7	1	1	1
2.25	0.88	0.95	1.65	149.6	1500	10.96	400	85.54	0	1	1
2.03	0.89	0.95	1.54	326	1330	5.31	1240	71.87	1	0	0
2.27	0.85	1.09	1.62	127.8	1000	8.33	100	94.14	1	1	1
2.09	1.01	0.72	1.17	982.7	2150	3.63	0	73.37	1	1	1
2.16	1.11	0.86	1.35	140	1200	6.21	215	88.87	1	0	1
2.2	0.79	1.04	1.44	110	1500	8.15	100	62.016	1	1	0
1.76	0.98	1.08	1.37	60.4	100	4.64	1400	67.39	1	0	1
2.37	0.67	0.85	0.42	260.9	800	6.27	50	71.06	0	1	1
2.03	0.68	1.06	1.21	582	1700	5.69	-20	71.89	1	1	1
2.24	1.03	0.98	1.59	234	2000	9.03	300	116.57	1	0	1
2.29	1.13	0.8	1.42	367.3	550	3.42	400	41.82	1	1	1
2.09	1.13	1.06	1.25	726.5	1100	4.61	0	100.69	1	0	1
2.11	0.81	0.62	1.95	1611.3	600	3.19	500	62	0	1	1
1.89	1.09	0.73	1.52	243.3	700	4.27	100	37.6	1	1	1
1.9	1.22	1.36	2.28	451.4	700	2.92	214	55.164	1	1	1
2.12	1.11	0.68	1.49	406.4	900	3.42	0	35.695	1	1	1
1.36	0.79	0.81	1.2	73	1800	9.97	-100	105.89	1	1	1
2.1	0.73	0.73	1.4	442.4	1100	6.4	1595	48.11	1	1	0
